# Ten Global “Hotspots” for the Neglected Tropical Diseases

**DOI:** 10.1371/journal.pntd.0002496

**Published:** 2014-05-29

**Authors:** Peter J. Hotez

**Affiliations:** 1 National School of Tropical Medicine at Baylor College of Medicine, Houston, Texas, United States of America; 2 Sabin Vaccine Institute and Texas Children's Hospital Center for Vaccine Development, Houston, Texas, United States of America; 3 James A. Baker III Institute at Rice University, Houston, Texas, United States of America

Since the founding of *PLOS Neglected Tropical Diseases* more than six years ago, I have written about the interface between disease and geopolitics. The neglected tropical diseases (NTDs) are the world's most common infections of people living in poverty [1]. Where they are widespread in affected communities and nations, NTDs can be highly destabilizing and ultimately may promote conflict and affect international and foreign policy [2]. Many of the published papers in this area were recently re-organized in a PLOS “Geopolitics of Neglected Tropical Diseases” collection that was posted on our website in the fall of 2012, coinciding with the start of our sixth anniversary [3]. From this information, a number of new and interesting findings emerged about the populations who are most vulnerable to the NTDs, including the extreme poor who live in the large, middle-income countries and even some wealthy countries (such as the United States) that comprise the Group of Twenty (G20) countries [4], as well as selected Aboriginal populations [5]. Together, the PLOS “Geopolitics of Neglected Tropical Diseases” collection and the G20 analyses identified more than a dozen areas of the world that repeatedly show up as ones where NTDs disproportionately affect the poorest people living at the margins. Here, I summarize what I view as ten of the worst global “hotspots” where NTDs predominate (Figure 1). They represent regions of the world that will require special emphasis for NTD control and elimination if we still aspire to meet Millennium Development Goals (MDGs) and targets by 2015; they are regions that may need to be highlighted again as we consider post-MDG aspirations and new Sustainable Development Goals (SDGs).

**Figure 1:**
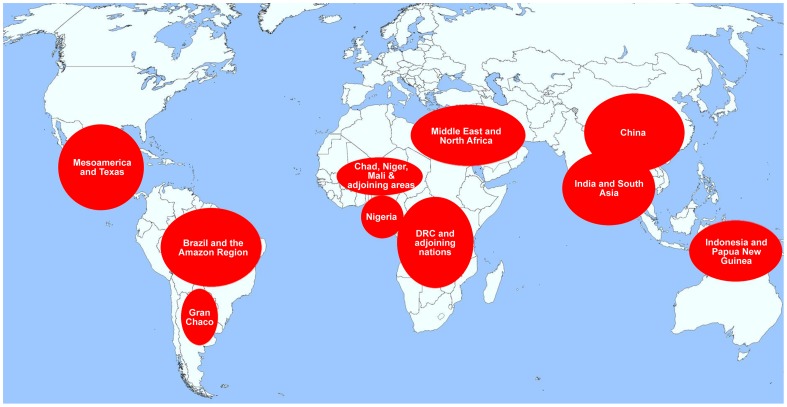
Ten neglected tropical disease “hotspots” around the globe.

## The Americas

In the Americas, there are at least three major zones where NTDs concentrate.

### Brazil and the Amazon Region

In 2008, I found that Brazil has the largest number of NTDs in the Western hemisphere; they are particularly common among the millions of Brazilians who live on less than US$2 per day [6]. Brazil has almost all of the cases of blinding trachoma, leprosy, and schistosomiasis in the Americas, as well as most of the visceral leishmaniasis, hookworm, and dengue, and one-half of the ascariasis cases [6]. Lymphatic filariasis (LF) and onchocerciasis also occur, and Brazil has the largest number of Chagas disease cases in the world—1.9 million cases—although transmission of Chagas disease in the country has been greatly diminished or even eliminated [7]. Poverty reduction measures constituted an important component of former President Luiz Inácio Lula da Silva's administration and are subsequently being continued by President Dilma Roussef [8]. These measures include efforts at NTD control; however, it remains unclear the extent to which the total NTD burden has diminished over the last five to six years. Also in Brazil and immediately beyond its border is the Amazon region shared among the nations of Brazil, Colombia, Peru, Venezuela, Ecuador, Bolivia, Guyana, Suriname, and French Guiana, with a substantial but as-still-yet-unmeasured number of NTD cases and disease burden from Chagas disease, vivax malaria, arbovirus infections, leishmaniasis, and intestinal helminthiases.

### Gran Chaco

Almost 10 million people inhabit the Gran Chaco, an area that spans eastern Bolivia, Paraguay, northern Argentina, and portions of two Brazilian states—Mato Grosso and Mato Grosso do Sul [9]. The area is an agriculturally intensive lowland region with a warm climate [9]. Among the major NTDs endemic to the region are intestinal helminth infections, including strongyloidiasis [10] and widespread Chagas disease [11] with triatomine insecticide resistance [12], although no disease burden information specifically for this region is available.

### Mesoamerica and Texas

Mesoamerica includes Mexico's poorest states in the southern region, such as Chiapas, Guerrero, and Oaxaca [13], and impoverished Central American countries, such as El Salvador, Guatemala, Honduras, and Nicaragua. In Mexico, approximately 11 million people live in extreme poverty, with intestinal helminth infections, cysticercosis, cutaneous leishmaniasis (CL), and dengue representing the most common NTDs [13], [14], in addition to at least 1 million cases of Chagas disease [7], [14]. These same diseases are also widespread among the 30% of the population who live in extreme poverty in Central America [15], including approximately 800,000 cases of Chagas disease [7]. Although Texas is not generally considered a part of the Mesoamerican region, there is evidence that cysticercosis, CL, dengue, and even Chagas disease are widespread in South Texas and even in parts of Houston, which is emerging as the first major city in the United States with serious NTDs [16]. According to one estimate, Chagas disease results in almost US$1 billion in economic losses annually in the US [17].

## Sub-Saharan Africa (SSA)

SSA has a high concentration of NTDs globally, accounting for approximately one-quarter to one-third of the world's cases of the three major intestinal helminth infections (namely, ascariasis, trichuriasis, and hookworm infection), more than one-third of the LF, one-half of the trachoma, and all or most of the schistosomiasis, onchocerciasis, loiasis, and human African trypanosomaisis (HAT) [18].

### Nigeria

I have referred to Nigeria as “ground zero” for the NTDs because it ranks first in SSA in terms of the number of cases of all three intestinal helminth infections, schistosomiasis, LF, and onchocerciasis [18], [19]. Following publication of this information, the government of Nigeria redoubled efforts to expand NTD control and elimination efforts [20].

### Democratic Republic of Congo (DRC) and Adjoining Nations: South Sudan, Central African Republic, Northern Uganda, and Angola

DRC ranks closely behind Nigeria in terms of the total number of NTD cases, ranking second or third in most of the NTD disease categories and first in HAT and leprosy [18], [21]. DRC is still recovering from the last quarter of the 20th century when it was known as Zaire, during the reign of Mobutu Sese Seku, which was accompanied by the re-emergence of HAT and other diseases [21]. However, DRC is not alone, as long-standing conflicts and public health infrastructure declines in neighboring South Sudan, Central African Republic, northern Uganda, and Angola, may make this part of SSA one of the most NTD-affected regions in the world [18], [22]. South Sudan, which became an independent state in 2011, will likely soon become the last country to eradicate guinea worm infection [18].

### Chad, Niger, and Mali and Adjoining Sahelian Areas

These three adjoining nations have also suffered from widespread conflict and NTDs in recent years, and with it high rates of trachoma, schistosomiasis, and intestinal helminth infections [18], [23]. The nations of Niger and Mali are representative of the problem of high rates of NTDs occurring among selected nations of the Organisation of the Islamic Conference—the world's Islamic countries [23]. Still another region for strong consideration is among the southern and eastern African countries of Mozambique, Malawi, Tanzania, and Zimbabwe, where female urogenital schistosomiasis and other NTDs are widespread.

## Asia and Oceania

The largest number of NTDs currently occurs in Asia, led by the large emerging market economies of India, Indonesia, and China [24]–[27].

### Indonesia and Papua New Guinea

Indonesia alone has approximately 10% of the world's cases of intestinal helminth infections, LF, and leprosy, in addition to more than one-half of the dengue deaths in Southeast Asia and a significant problem with other arbovirus infections and yaws [24]. Neighboring Papua New Guinea also accounts for most of the cases of hookworm infections and LF in Oceania, in addition to large numbers of cases of yaws and scabies, trachoma, leprosy, balantidiasis, and cholera outbreaks [25].

### India and South Asia

Nearly one-half or more of the cases of visceral leishmaniasis, LF, and leprosy occur in India and South Asia, in addition to one-third of the rabies deaths, one-quarter of the cases of intestinal helminth infections, and a massive but still ill-defined burden of disease from dengue and Japanese encephalitis [26].

### China

Rapid economic growth in eastern China has left behind high levels of disease and poverty in China's southwestern provinces of Sichuan, Guizhou, and Yunnan, where some of the highest rates of intestinal helminth infections are found [27]. China has the largest number of cases of the food-borne trematode infections, clonorchiasis, and paragonomiasis occurring in Guangdong Province in the South and some northern provinces, while more than 500,000 cases of schistosomiasis occur along the Yangtze River and its tributaries [27]. Trachoma and leprosy still occur [27]. A recent Global Burden of Disease analysis for China found that the NTDs are responsible for 3.7 million disability-adjusted life years (DALYs) lost annually, more than the DALYs lost from HIV/AIDS and tuberculosis [28].

## The Middle East

Approximately 65 million people live on less than US$2 per day in the Middle East and North Africa. These impoverished individuals suffer from high rates of intestinal helminthiases, LF, schistosomiasis, fascioloiasis, leishmaniasis, leprosy, and trachoma [29]. Overall, the highest rates of NTDs are found in Egypt and Yemen, but there are also a significant number of NTDs in Iran, Algeria, and elsewhere [28].

## Concluding Comments

These ten areas exhibit some of the world's highest concentrations of NTDs, although they vary with respect to having a modest prevalence among a large population versus hyperendemicity among a smaller population. Of interest is the finding that, with the important exception of SSA, they mostly include middle-income countries and nations belonging to the G20 [4]. The hotspot areas represent regions that require intensified efforts for NTD control and elimination, which would include access to essential NTD medicines through mass drug administration (also known as preventive chemotherapy), but also vector management and control. In a previous paper I pointed out the opportunities for the G20 countries to engage in scientific research and development to produce new drugs and vaccines affecting their regions, and possibly diplomacy to promote international scientific cooperation [4]. These regions would comprise key areas to target as a means to achieve the MDGs and to help set goals and targets past 2015 and for the new SDGs [30], [31].

This list represents a personal view of what I consider some of the most important NTD-affected areas in the world. I welcome comments and opinions from the NTD community on other regions and parts of the world I might have missed or where the readers believe there should be renewed emphasis for control and elimination.
